# Drotrecogin alfa (activated) in severe sepsis: a systematic review and new cost-effectiveness analysis

**DOI:** 10.1186/1471-2253-7-5

**Published:** 2007-06-25

**Authors:** Vania Costa, James M Brophy

**Affiliations:** 1 Technology Assessment Unit, McGill University Health Centre, Montreal, Quebec, Canada; 2 Department of Medicine, McGill University Health Centre, Montreal, Quebec, Canada

## Abstract

**Background:**

Activated drotrecogin alfa (human activated protein C, rhAPC), is produced by recombinant DNA technology, and purports to improve clinical outcomes by counteracting the inflammatory and thrombotic consequences of severe sepsis. Controversy exists around the clinical benefits of this drug and an updated economic study that considers this variability is needed.

**Methods:**

A systematic literature review was performed using Medline, Embase and the International Network of Agencies for Health Technology Assessment (INAHTA) databases to determine efficacy, safety and previous economic studies. Our economic model was populated with systematic estimates of these parameters and with population life tables for longer term survival information. Monte Carlo simulations were used to estimate the incremental cost-effectiveness ratios (ICERs) and variance for the decision analytic models.

**Results:**

Two randomized clinical trials (RCTS) of drotrecogin alfa in adults with severe sepsis and 8 previous economic studies were identified. Although associated with statistical heterogeneity, a pooled analysis of the RCTs did not show a statistically significant 28-day mortality benefit for drotrecogin alfa compared to placebo either for all patients (RR: 0.93, 95% CI: 0.69, 1.26) or those at highest risk as measured by APACHE II ≥ 25 (RR: 0.90, 95% CI: 0.54, 1.49). Our economic analysis based on the totality of the available clinical evidence suggests that the cost-effectiveness of drotrecogin alfa is uncertain (< 59% probability that incremental cost-effectiveness ratio (ICER) life year gained (LYG) ≤ $50,000/LYG) when applied to all patients with severe sepsis. The economic attractiveness of this therapy improves when administered to those at highest risk as assessed by APACHE II ≥ 25 (93% probability ICER ≤ $50,000/LYG) but these results are not robust to different measures of disease severity.

**Conclusion:**

The evidence supporting the clinical and economic attractiveness of drotrecogin alfa is not conclusive and further research appears to be indicated.

## Background

Sepsis is a complex syndrome with protean etiologies characterized by a systemic inflammatory and procoagulant response to an infection, and is considered severe in the presence of acute organ dysfunction [1]. Endogenous protein C activation attempts to counteract these manifestations of sepsis and drotrecogin alfa, a form of human activated protein C produced by recombinant DNA techniques [2] has been approved for the subgroup of patients with severe sepsis and the highest risk of death [3-5].

Controversy has plagued this drug since its 2001 FDA evenly divided approval vote [6,7]. This controversy emanates from approval being based on a single randomized controlled trial (RCT) (PROWESS) and particularly on one subgroup analysis. Moreover concerns exist regarding drotrecogin alfa's cost-effectiveness and the inconsistent results observed in more recent studies. Several economic analyses have nevertheless suggested that the drug may be cost-effective but these early studies have generally not considered the totality of the efficacy evidence now available. Therefore we performed a systematic review of all published drotrecogin alfa evidence, as a prelude for an updated economic analysis to assist in difficult resource allocation decisions.

## Methods

### Literature search

A systematic literature search of RCTs and economic evaluations comparing drotrecogin alfa and placebo in adult and pediatric patients published in English or French was performed using Medline and Embase databases (search (MeSH) terms: (Drotrecogin OR Activated protein C OR Xigris) AND (Sepsis). The search included publications until Dec 31 2006. The reference lists of the publications identified were also searched for additional relevant publications.

### Methods for economic analysis

**Figure 1:**
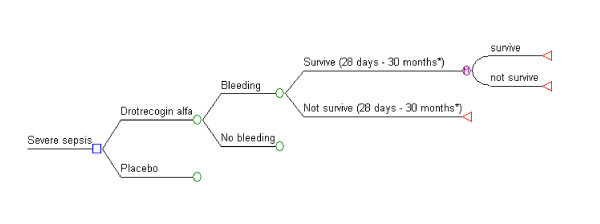
**Simplified decision analytic model employed**. *The individual survival probabilities between 28 days and 30 months were included in the model but are not shown in this figure. A similar tree was used in the bleeding and non-bleeding arms, and for the drotrecogin alfa and placebo arms (not shown). After the 30 months a Markov model was employed (determined by the node M).

Cost-effectiveness analysis ($/life year gained (LYG) was performed using a decision-tree model (see Figure 1) and a 20 year horizon, based on the approximate life-expectancy of our base case patient population. Drotrecogin alfa efficacy measures for our economic model were ascertained, when possible, by combining RCTs results from our systematic review in a random effects meta-analysis (RevMan V 4.2, Cochrane Collaboration, Oxford, England). Beta distributions for model parameters were derived from this information.

Survival rates for the first 30 months from RCT(s) were available for drotrecogin alfa and placebo from the PROWESS study [8]. We assumed that this numerical difference in survival at 30 months persisted without any further increases or decreases in long term survival. Long term survival could be estimated from population life tables, corrected for the higher mortality risk in sepsis patients. Costs beyond three years were also considered to be similar between the two groups. Separate models were developed for the whole study population and the subgroups at highest risk of death. Complication rates were taken from a pooled analysis using data available from the RCTs.

Probabilistic sensitivity analyses using 10,000 Monte Carlo simulations were used to estimate the incremental cost-effectiveness ratios (ICERs) and variance (TreeAge Pro 2007). The economic analyses were performed from the public healthcare provider viewpoint. Multiple sensitivity analyses were performed using a different clinical outcome (quality-adjusted life-years (QALYs) with utilities measured with the EuroQoL-5D questionnaire [9], as well as varying time horizons and discount rates. Table 1 shows the parameters and characteristics of the models employed. Costs are provided in 2006 US dollars.

**Table 1:** Parameters used in our decision analytic models.

Parameter	Base case	Source	Univariate Sensitivity analyses
**Effectiveness measure**	Life-years gained	Based on long-term survival	Quality-adjusted life years (QALYs)
**Groups**	All patients APACHE II ≥ 25	-	-
**Mean entry age**	60 years	PROWESS [13] and ADDRESS [14]	-
**Life-expectancy for a 60-year old**	Males: 20 years Females: 24 years	Statistics Canada Life-tables[59]	-
**Time horizon**	20 years	Statistics Canada Life-tables [59]	30 months to 30 years
**Discount rate**	3%	-	0, 5%
**Perspective of the analyses**	Public health care provider	-	-
**Survival rates (see Tables 4-6 for details)**	Short-term (28-days)	PROWESS [13] and ADDRESS [14] RCTs	Probabilistic sensitivity analysis using a beta distribution defined by the point estimate and variance from RCTs for each group.
	Mid-term (hospital discharge – 30 months)	Long-term PROWESS [8] RCT	
	Long-term beyond 30 months	Observational study in severe sepsis patients [40] and Canadian life table (2000–2002) [59] adjusted for a higher mortality in severe sepsis patients*	
**Complication rates**	28-day bleeding rates	PROWESS [13] and ADDRESS [14]	-
Resources included in the cost analyses**	Drug acquisition	Pharmacy department MUHC	-
	Hospitalization for the sepsis episode	Canadian long-term observational study in severe sepsis patients [40]	
	Treatment complications Years 1–3 follow-up healthcare treatment costs		

* The lifetime annual survival rates in the general population were adjusted for a higher severity in severe sepsis patients according to the absolute difference in mortality between the age-specific survival in the general population (1.1% for a 63-year old) [59] and that of a 3-year Canadian long-term observation study in severe sepsis patients (4.2% and 6.2% in all patients and those with APACHE II ≥ 25 respectively at 3 years, mean age at cohort entry: 61.1 years [40]).

** Costs associated with the severe sepsis episode incurred after three years were not available and were considered identical for the two groups.

Our cost analysis included direct costs such as hospitalization, emergency room visits, day-surgery, and physician charges [40].

The presence of overlapping distributions in the effectiveness, i.e., non statistically significant or small differences between two comparators, or even negative effectiveness, results in instability in the calculation of ICERs and their confidence intervals thereby rendering their interpretation difficult [10,11]. Therefore we have graphically presented the results from our probabilistic sensitivity analyses in the cost-effectiveness plane as this shows the proportion of simulations where drotrecogin alfa had a higher effectiveness and/or cost compared to placebo. Acceptability curves using net health benefits are also presented as this measure overcomes the difficulties in interpreting negative ICERs [12].

## Results

### Systematic literature review

**Figure 2:**
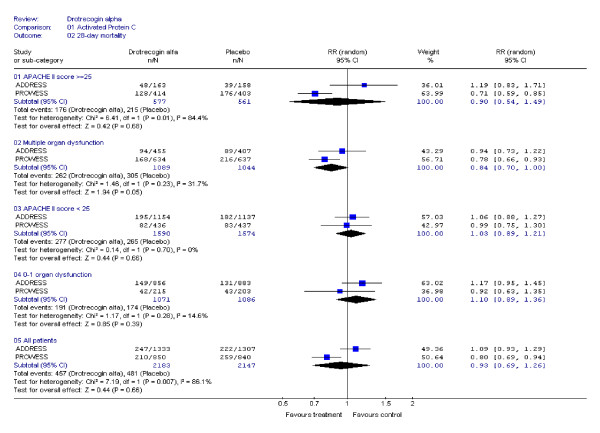
**28-day mortality meta-analysis**. Some of the numbers in the graph are approximations as they were derived from figures in the published studies.

**Figure 3:**
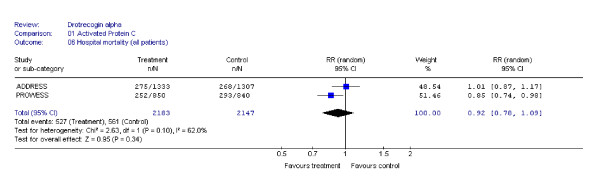
**In-Hospital mortality meta-analysis**. Some of the numbers in the graph are approximations as they were derived from figures in the published studies.

Our systematic literature search identified 2 adult randomized, double-blind, placebo-controlled trials [13,14] (see Table 2) with long-term results available only for one [8]. Both RCTs were terminated prematurely, one for efficacy [13] and the other for futility [14]. Although the disease severity criteria for these two trials were slightly different, the pooling of these studies is justified since the same disease entity is being studied with the same research design using the same treatment protocol (see Table 2). The pooled 28-day efficacy results are shown in Figure 2. The totality of the available evidence indicates no statistically significant 28-day mortality reduction for drotrecogin alfa in patients with severe sepsis (RR, 0.93, 95% CI: 0.69, 1.26), even when stratified by disease severity (APACHE II ≥ 25, RR: 0.90, 95% CI: 0.54, 1.49). Despite the relative homogeneity in the 2 populations, there was presence of statistical heterogeneity in the overall results. However when the analyses were performed using the number of failed organs as a measure of disease severity or when in-hospital mortality was used as outcome (Figure 3), there was no longer evidence of statistical heterogeneity between the two RCTs.

**Table 2:** Characteristics of available adult RCTs

	**PROWESS **[13]		**ADDRESS **[14]	
Inclusion criteria summary	Severe sepsis ≥ 1 organ dysfunction*		Severe sepsis ≥ 1 organ dysfunction* and low risk of death	
Therapy initiation	within 48 hours of first organ dysfunction		Within 48 hours of first organ dysfunction	
Active treatment	96 hour intravenous infusion of drotrecogin alfa (24 μg/kg/hr)		96 hour intravenous infusion of drotrecogin alfa (24 μg/kg/hr)	
Placebo	96-hour intravenous infusion (0.9% NaCl)		96-hour intravenous infusion (0.9% NaCl)	

Baseline characteristics	Drotrecogin alfa	Placebo	Drotrecogin alfa	Placebo
N	N = 850	N = 840	N = 1333	N = 1307
Age, mean ± SD (years)	60.5 ± 17.2	60.6 ± 16.5	58.8 ± 16.8	58.6 ± 16.7
Male (%)	56.1%	58%	56.3%	58.5%
APACHE II score, mean ± SD	24.6 ± 7.6	25 ± 7.8	18.2 ± 5.8	18.2 ± 5.9
Mechanical ventilation (%)	73.3%	77.6%	56.3%	55.8%
Shock (%)	70.4%	71.7%	NA	NA
Use of any vasopressor (%)	71.8%	75.5%	47.9%	47.5%
≥ 2 organ dysfunctions (%)	74.6%	75.8%	34.5%	31.5%
Time from 1^st ^organ dysfunction to study drug start (hours), mean ± SD	17.5 ± 12.8	17.4 ± 9.1	22.5 ± 13.6	22.6 ± 13.8

* Cardiovascular, renal, respiratory, hematologic, or unexplained metabolic acidosis

RCT = randomized controlled trial/SD = standard deviation

Survival beyond 28 days or hospital discharge has been reported only for the PROWESS study [8]. No statistically significant mortality differences were observed in follow-up from 3 to 30 months in the whole cohort of patients and most reported subgroup analyses [8] except for the subgroup defined by an Acute Physiology and Chronic Health Evaluation (APACHE) II [15] score ≥ 25 (30-month survival: 45.6% vs 33.8% for drotrecogin alfa and placebo respectively, p = 0.001) [8]. In contrast, in the subgroup with ≥ 2 organ dysfunctions, which was the criterion used by the regulatory agency of the European Union to define higher disease severity [4], there was no statistically significant survival benefit with drotrecogin alfa over placebo [8], the same in other subgroups.

Because of its antithrombotic and profibrinolytic effects, bleeding complications may be anticipated with drotrecogin alfa [16]. Among the RCTs identified, the PROWESS reported 30 (3.5%) 28-day serious bleeding events with drotrecogin alfa and 17 (2%) with placebo (p = 0.06), in the ADDRESS study there were 51 (3.9%) and 28 (2.2%) such events respectively (p = 0.01) [14]. Pooling these two studies together using the inverse variance method yielded a 3.7% (± 1.06) rate of 28-day serious bleeding events with drotrecogin alfa and 2.1% (± 1.04) with placebo.

Despite only 2 RCTs, there were numerous secondary publications [17-30] involving subgroup analyses based on the PROWESS [13] trial. In addition 3 meta-analyses [31-33], and 9 economic analyses [34-42] were identified (Table 3). The calculated cost/life-year gained (LYG) for all patients varied between $US 8,500 and $US 33,300 and was generally slightly lower for higher risk patients (Table 3). However, most of these analyses modeled long term results by assuming that the PROWESS 28-day mortality results were sustained and durable which has been shown to be incorrect (see above).

**Table 3:** Summary of the economic studies identified in the literature (values in US$). Results in all patients and by severity subgroups when available, drotrecogin alfa compared to placebo

*Study Country (year of publication)*	Incremental Cost*	Incremental * effectiveness (LYG)	ICER* All severe sepsis patients	ICER* Higher risk patients
Manns et al. [40] Canada (2002)	-	0.38 LYG 0.76 LYG (APACHE II ≥ 25)	$ 27,936/LYG	$ 19,726/LYG (APACHE II ≥ 25)
Neilson et al [39] Germany (2003)	$10,533 (all patients) $11,238 LYG (≥ 2 organ dysfunctions)	0.47 LYG 0.69 LYG (≥ 2 organ dysfunctions)	US$ 22,411/LYG (3% discounting)	$ 17,419/LYG (≥ 2 organ dysfunctions)
Angus et al. [41] US (2003)	$16,000 (±4,200) (all patients)	0.48 LYG (SD 0.29) (all patients)	$ 33,300/LYG	-
Betancourt et al [38] US (2003)	$6,246 (all patients) $6,246 (≥ 2 organ dysfunctions)	0.06 lives saved** 0.08 (≥ 2 organ dysfunctions)	$ 104,100/life saved**	$78,075/life saved (≥ 2 organ dysfunctions)**
Fowler et al. [34] Canada (2003)	$10,745 (all patients) $15,166 (APACHE ≥ 25)	0.68 LYG 1.4 LYG (APACHE ≥ 25)	$ 15,801/LYG	$ 10,833/LYG (APACHE ≥ 25)
Riou França et al. [35] France (2006)	$7,545 (all patients) $7,333 (2 organ dysfunctions)	0.64 LYG (all patients) 0.57 (2 organ dysfunctions)	$ 11,812/LYG	$12,942/LYG (2 organ dysfunctions)
Hjelmgren et al [36] Sweden (2005)	$12,272 (all patients) $14,663 (≥ 2 organ dysfunctions)	0.544 LYG 0.474 LYG (≥ 2 organ dysfunctions)	US$ 22,920/LYG	$ 30,853/LYG (≥ 2 organ dysfunctions)
Davies et al. [37] UK (2005) Using PROWESS data	$9,517	1.12 LYG	US$ 8,533/LYG	-
Green et al. [42] UK (2006)	$11,645 (SD $1,098) (all patients) $12,336 (SD $1,430) (≥ 2 organ dysfunctions)	1.144 LYG (SD 0.34) 1.351 LYG (≥ 2 organ dysfunctions)	$ 10,176/LYG	$ 9,132/LYG (≥ 2 organ dysfunctions)

LYG = life-year gained/SD = standard deviation/TA = technology assessment

*Results comparing drotrecogin alfa to placebo

** $/LYG not available.

### Current economic analysis

**Figure 4:**
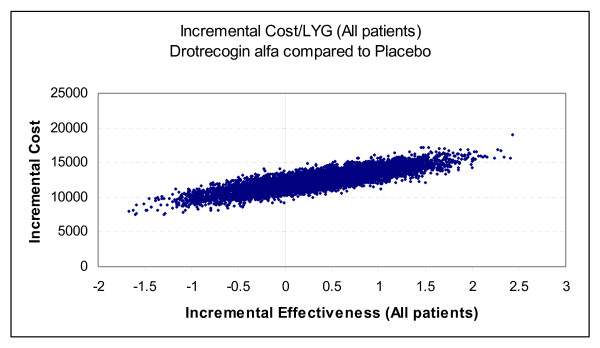
**Cost-effectiveness plane for all patients – 10,000 Monte Carlo simulations (20-year time horizon, 3% discounting)**. The points to the left of the vertical line correspond to a lower efficacy and higher cost with drotrecogin alfa compared to placebo.

**Figure 5:**
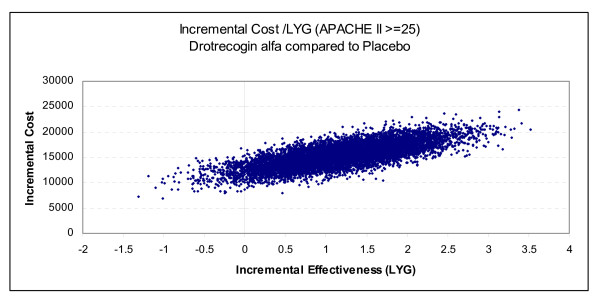
**Cost-effectiveness plane for patients with APACHE II ≥ 25 – 10,000 Monte Carlo simulations (20-year time horizon, 3% discounting)**. The points to the left of the vertical line correspond to a lower efficacy and higher cost with drotrecogin alfa compared to placebo.

Our decision analytic model is shown in Figure 1 and the parameter and cost estimates are given in Tables 4, 5, 6. The totality of the evidence for all patients revealed considerable overlapping of the survival estimates in treated and placebo patients resulting in approximately 27% of the simulations with a negative ICER (i.e. more expensive but less effective) for drotrecogin alfa compared to placebo (see Figure 4). The model using APACHE II >= 25 as a severity measure showed a lower chance, approximately 3%, of a negative ICER (Figure 5).

**Table 4:** Cumulative survival parameter estimates with all patients with severe sepsis

Drotrecogin alfa			
**Cumulative survival**	**PROWESS**	**ADDRESS**	**Point estimate Pooled results (± SD)**

28-day	75.3% [13]	81.5% [14]	79.4% (± 1.06)
Hospital discharge	70.3% [8]	79.4% [14]	76.4% (± 1.11)
3-month	66.1% [8]	NA	66.1% (± 1.86)
6-month	62.2% [8]	NA	62.2% (± 1.97)
12-month	58.9% [8]	NA	58.9% (± 2.05)
30-month	52.6% [8]	NA	52.6%(± 2.10)

**Placebo**			

**Cumulative survival**	**PROWESS**	**ADDRESS**	**Pooled results (± SD)**

28-day	69.2% [13]	83% [14]	78.8% (± 1.04)
Hospital discharge	65.1% [8]	79.5% [14]	74.95% (± 1.12)
3-month	62.4% [8]	NA	62.4% (± 1.93)
6-month	60.3% [8]	NA	60.3% (± 1.98)
12-month	57.2% [8]	NA	57.2% (± 2.06)
30-month	49.3% [8]	NA	49.3% (± 2.10)

CI = confidence interval/NA = not available/SA = sensitivity analysis/SD = standard deviation

**Table 5:** Cumulative survival parameter estimates in patients with APACHE II >= 25

Drotrecogin alfa			
**Cumulative survival**	**PROWESS**	**ADDRESS**	**Point estimate Pooled results (± SD)**

28-day	69.1% [2]	70.5%[14]	69.45% (± 3.55)
3-month	58.9% [8]	NA	58.9% (± 2.42)
6-month	55.2% [8]	NA	55.2% (± 2.44)
12-month	52.1% [8]	NA	52.1% (± 2.46)
30-month	45.6% [8]	NA	45.6% (± 2.45)

**Placebo**			

**Cumulative survival**	**PROWESS**	**ADDRESS**	**Pooled results (± SD)**

28-day	56.3% [2]	75.3% [14]	62.93% (± 3.40)
3-month	48.4% [8]	NA	48.4% (± 2.49)
6-month	45.3% [8]	NA	45.3% (± 2.48)
12-month	41.3% [8]	NA	41.3% (± 2.45)
30-month	33.8% [8]	NA	33.8% (± 2.36)

CI = confidence interval/NA = not available/SA = sensitivity analysis/SD = standard deviation

* Calculated according to the information provided in the studies.

**Table 6:** Cost estimates used in the decision analytic models. Costs in US dollars.

Costs	Model with all patients	Model in patients with APACHE II score >= 25	Source
Drug costs * (drotrecogin alfa)	$9,700	$9,700	MUHC (Pharmacy department)
Bleeding episode costs	$12,090	$12,090	Manns et al [40]
Hospitalization costs (severe sepsis episode)	$47,960	$51,095	Manns et al. [40]
1-year costs (after hospital discharge)**	$20,641	$29,879	Manns et al. [40]
Year 2 costs**	$6,641	$8,083	Manns et al. [40]
Year 3 costs**	$6,290	$5,762	Manns et al. [40]

* Drug costs refer to acquisition costs of drotrecogin alfa and were therefore used only in this group. Other costs were assumed to be identical in both groups.

Costs from the article by Manns et al. [40] were converted to Canadian dollars according to the exchange rate used in the article (US$1 = CDN$1.47) and adjusted for inflation according to Bank of Canada rates.

** Costs with 3% discounting

This included direct health care costs for hospitalizations, emergency visits, day surgeries, and physicians' costs [40].

**Figure 6:**
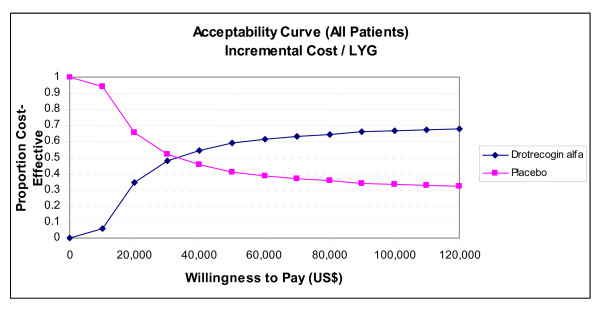
**Acceptability curve (incremental cost/LYG). Lifetime decision model (all patients, 3% discounting, 20-year time horizon)**. LYG = life-years gained.

**Figure 7:**
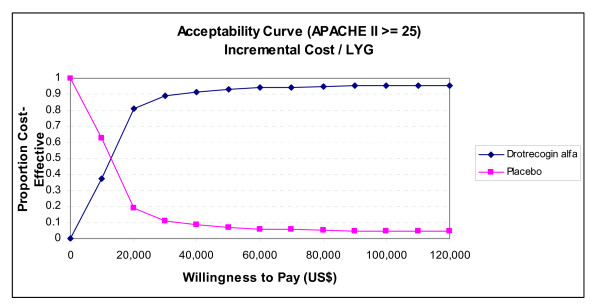
**Acceptability curve (incremental cost/LYG). Lifetime decision model (high risk patients with APACHE II = 25, 3% discounting, 20-year time horizon)**. LYG = life-years gained.

Due to this instability in ICERs, we have elected to concentrate on acceptability curves that show the proportional benefit at varying willingness to pay (WTP) thresholds. Figures 6 and 7 show the net benefits acceptability curve obtained for all patients and those with APACHE II ≥ 25 respectively. In the model for all patients, there was a 48% chance that the ICER will be ≤ $30,000/LYG, and 59% chance that it will be ≤ $50,000 (Figure 6). Considering only those patients with APACHE II ≥ 25, there is an 89% chance of an ICER ≤ $30,000/LYG, and a 93% chance of an ICER ≤ $50,000/LYG (Figure 7). Since drotrecogin alfa had a higher cost compared to placebo due to its acquisition costs, the drug was never the dominant strategy (defined by a higher effectiveness and lower costs) in our analyses. Table 7 presents the incremental cost and life-years gained with drotrecogin alfa compared to placebo in our models.

**Table 7:** Incremental effectiveness and costs from our base-case analyses (drotrecogin alfa compared to placebo)

*Model*	*Incremental Cost (95% CI)*	*Incremental effectiveness (LYG)*	*% simulations where drotrecogin alfa has a lower effectiveness than placebo*
All patients	$11,024 ($8,670, $13,341)	0.344 (-0.807, 1.476)	27%
APACHE II ≥ 25	$13,612 ($9,785, $17,421)	1.191 (-0.082, 2.417)	3%

CI = confidence interval

LYG = life-years gained

Sensitivity analyses using QALYs as the measure of effectiveness did not change the results appreciably. Similar results were obtained with varying time horizons (10–30 years) and discount rates (data not shown). Importantly in the high risk group when defined by ≥ 2 organ system failures the probability that the ICER for drotrecogin alfa compared to placebo was ≤ $50,000 was only 52%, testifying to the lack of robustness in this subgroup economic analysis.

## Discussion

Our systematic review identified 2 RCTs, 3 meta-analyses, and 9 economic analyses comparing drotrecogin alfa and placebo in adults. Our results question the short term survival benefit of drotrecogin alfa and are in agreement with two previous meta-analyses [31,32]. While much clinical attention has been previously focused on the mortality benefit seen in the PROWESS APACHE II ≥ 25 subgroup, our analysis shows that when the totality of the evidence is examined uncertainty exists as to this benefit. Supporting this conclusion is the lack of benefit in the high risk group when assessed by a different measure (≥ 2 organs dysfunction) and diminishing benefits over time. Due to the difficulties in determining the APACHE II scores [15], the European Union regulatory authorities have preferred multiple organ dysfunction as a measure of disease severity and for labeling indications [43]. Other investigators have also failed to demonstrate any survival advantage with drotrecogin alfa at hospital discharge in other high risk groups, including the need for vasopressor support, APACHE II score between 30 and 53, and protein C deficiency [28]. The multiple subgroup analyses, both pre-specified and not, of the PROWESS data [17-30] increases the possibility that the APACHE II subgroup results may represent a false positive finding.

In contrast to our results, a company-sponsored meta-analysis has reported a statistically significant reduction in the 28-day all-cause mortality in all patients with drotrecogin alfa compared to placebo [33]. However, this included studies involving indirect comparisons with different sepsis drugs and without contemporaneous controls [33]. Variations in patient entry criteria, treatments received, lack of randomization, residual confounding and the potential for calendar time bias undermines the validity of these results.

Our economic analyses showed that drotrecogin alfa may not be cost-effective in all sepsis patients due to uncertainties in the survival benefits when all available evidence is considered. Our economic conclusions differ from most previous publications, which have assumed that the large 28-day mortality advantage in PROWESS would be sustained and which has not been confirmed either in a long term follow-up or in another RCT. Our conservative estimate of cost-effectiveness, while in contradiction to other published analyses, nevertheless more adequately models the known long-term efficacy data for this drug.

Although the cost effectiveness of drotrecogin alfa improves when restricted to treating those at the highest risk, caution must be exercised in attempting to justify treatment only to a specific subgroup. Severe sepsis is a very complex syndrome [44], therefore, even in the RCTs it cannot be ruled out that some of the multiple measured and unmeasured baseline confounders may be unequally balanced with repeated subgroup analyses. Basing treatment on disease severity is problematic for several other reasons; not the least that the pooled data do not show a statistical benefit for very high risk, whether assessed by APACHE II or multiple organ failure score; second, APACHE II does not take into account important measures of severity in severe sepsis patients such as white blood cell count, number of days in hospital and ICU before the diagnosis of severe sepsis. [15].

The main limitation of our economic analyses centers on the uncertain estimate of clinical efficacy. While this been approached systematically the short term results are derived from only 2 trials with evidence of statistical heterogeneity between them and the long term results come from a single study. However as we have argued, the clinical similarities between the patient populations as well as the standardized active therapy suggests that the best estimate of clinical efficacy arises from the pooled estimate. Moreover the robustness of our model in different sensitivity analyses provides further assurance.

## Conclusion

The debate on the place of drotrecogin alfa persists as judged by the numerous recently published editorials and comments [6,7,43,45-58]. Hopefully our systematic and transparent economic model will assist clinicians and decision makers in assessing the current efficacy of drotrecogin alfa and its cost-effectiveness. At present, our analysis does not support the cost-effectiveness of drotrecogin alfa in all severe sepsis patients but suggests that a targeted approach to the very high risk patients may be appropriate while awaiting additional evidence.

## Abbreviations

APACHE – acute physiology and chronic health evaluation

CI – confidence interval

FDA – Food and Drug Administration

ICER – incremental cost-effectiveness ratio

LYG – life-years gained

QALY – quality-adjusted life-years

RCT – randomized control trials

RR – relative risk

WTP – willingness to pay

## Competing interests

The author(s) declare that they have no competing interests.

## Authors' contributions

JB designed the study. VC conducted the systematic literature search, reviewed the studies identified, and extracted the data from the eligible studies. JB reviewed the eligibility of the studies identified, and the data extraction. JB and VC carried-out the meta-analyses, economic evaluations and economic modeling. JB and VC prepared the manuscript. All authors read and approved the manuscript.

## Pre-publication history

The pre-publication history for this paper can be accessed here:

http://www.biomedcentral.com/1471-2253/7/5/prepub
